# Application of noncollapsing methods to the gene-based association test: a comparison study using Genetic Analysis Workshop 18 data

**DOI:** 10.1186/1753-6561-8-S1-S53

**Published:** 2014-06-17

**Authors:** Tian-Xiao Zhang, Yi-Ran Xie, John P Rice

**Affiliations:** 1 Department of Psychiatry, Washington University, 660 S. Euclid Ave., St. Louis, MO 63110, USA

## Abstract

Rare variants have been proposed to play a significant role in the onset and development of common diseases. However, traditional analysis methods have difficulties in detecting association signals for rare causal variants because of a lack of statistical power. We propose a two-stage, gene-based method for association mapping of rare variants by applying four different noncollapsing algorithms. Using the Genome Analysis Workshop18 whole genome sequencing data set of simulated blood pressure phenotypes, we studied and contrasted the false-positive rate of each algorithm using receiver operating characteristic curves. The statistical power of these methods was also evaluated and compared through the analysis of 200 simulated replications in a smaller genotype data set. We showed that the Fisher's method was superior to the other 3 noncollapsing methods, but was no better than the standard method implemented with famSKAT. Further investigation is needed to explore the potential statistical properties of these approaches.

## Background

During the past five years, genome-wide association studies (GWAS) have rapidly become a standard method for discovering susceptible genes for a variety of complex diseases [1]. Up to now, hundreds of loci with more than 3000 single-nucleotide polymorphisms from approximately 7000 GWAS have been reported to be associated with complex diseases [2]. Nevertheless, a large proportion of heritability is left unexplainable from GWAS results that are mainly based on association signals captured by common variants [3]. One potential explanation for this "missing heritability enigma" has been the contribution of rare variants, which is often not assessed in regular GWAS studies [3]. Unfortunately, traditional methods often fail in association mapping of rare variants because of poor statistical power. Several methods have been proposed to detect association signals for rare variants with improvements in statistical power compared to traditional methods [4-6].

As part of Genetic Analysis Workshop 18 (GAW18), simulated phenotypic data, based on a real sequencing data set, were provided to the scientific community to evaluate and compare statistical genetic methods for rare variants association mapping. We consider a two-stage, gene-based method to detect association signals for both common and rare variants. We first obtain significance *p *values by fitting a mixed effects model for each variant, and then apply 4 noncollapsing algorithms to obtain the gene-wise association *p *values. Collapsing (or burden) methods combine variant information by assuming consistent direction of effects across variants. None of the methods considered here adopt this assumption, although some (Fisher's, Gene Set Enrichment Analysis [GSEA], sequence kernel association test [SKAT]) do combine variant information.

## Methods

### Model fitting and algorithms

A mixed linear model was fitted for each variant as described in previous literature [7]. The model was defined as:

Y=Xβ+Qv+Zμ+ϵ

where Y is the quantitative trait of interest (we used first-visit systolic blood pressure [SBP] data were used in this study); × is the genotype; β is the fixed effects of the genotypes; and Q represents the population structure variables. In this study, we chose the first 10 principal components from principal component analysis (PCA) for *Q*;ν is the fixed effects of *Q*;*Z *is the variable that evaluates familial relatedness (the theoretical kinship matrix was used for *Z*); and *µ *is the random effects coefficient for *Z *that corrects the polygenic impact.

After obtaining the variant-wise *p *values by fitting the mixed linear model, four noncollapsing algorithms were modified and applied to the data set to obtain the gene-wise association *p *values. The algorithms of the 4 methods are summarized as followed:

1. Naïve method. The most significant variant-wise *p *values within a specific gene were chosen as the gene-wise association *p *values.

2. Fisher's method [8]. The gene-wise statistics were calculated through the following equation:

$$X=-2\overset{k}{\underset{i=1}{\sum }}{\text{log}}_{e}\left(p_{i}\right)$$

where *p_i _*is the *p *value for variant *i*, and *k *is the total number of variants within a specific gene. Because many variants are highly correlated, the basic assumption of independent tests for Fisher's method is violated. Fisher's formula will not have a chi-square distribution, so we assessed the significance via permutation analysis.

3. Simes' method [9]. The gene-wise *p *value was summarized by the following equation:

$$P_{simes}=\underset{i}{\min}\left\{\frac{kp_{i}}{i}\right\}$$

where *p_i _*is the *p *value for variant *i*, and *k *is the total number of variants within a specific gene.

4. GSEA method [10,11]. The test statistics (indicated as ES score) were aggregated from variant-wise *p *values within each gene via a Kolmogorov-Smirnov-like process in which running sums are accumulated. The equation is given as:

$$ES\left(S\right)=\underset{1\leq j\leq N}{\text{max}}\left\{\underset{G_{j*\epsilon S},j*\leq j}{\sum }\frac{|r_{\left(j*\right)}{|}^{p}}{N_{R}}-\underset{G_{j*\notin S},j*\leq j}{\sum }\frac{1}{N-N_{H}}\right\}$$

where *N *is the total number of variants, *r(j) *is the *j*^th ^largest statistic values, *N_H _*is the variant number of a given gene, *S *is any given gene, P is the parameter that gives a higher weight to variants with extreme statistic value, arbitrarily set to 1 in this study, and *N_R _*is given by:

$$N_{R}=\underset{G_{j*\epsilon S}}{\sum }|r_{\left(j*\right)}{|}^{p}$$

Statistical significance and adjustment for multiple hypothesis testing were assessed by a 1000-permutation-based procedure. A family-wise error rate (FWER) procedure was used to adjust for multiple hypothesis testing. The FWER is a highly conservative correction procedure that seeks to ensure that the list of reported results does not include even a single false-positive gene. In this study, the FWER *p *value was calculated as the fraction of all permutations whose highest statistics (or smallest *p *values) in all genes is higher than a given gene. In addition to the 4 noncollapsing algorithms introduced above, we also included 2 standard rare variants analysis methods: SKAT [12] and famSKAT [13] in our analysis. FamSKAT is an extended version of SKAT and can analyze rare variant when family correlations are present. Furthermore, to evaluate the statistical power of these methods, we extracted the variant information related to the 22 true-positive genes located on chromosome 3 and analyzed these data for all 200 simulated phenotype replicates.

### Data and computation

The chromosome 3 sequencing data were analyzed only for phenotype replicate number 1 because of a huge computational burden. The sequencing data were annotated by ANNOVAR[14]. Intergenic variants (variants at least 1 kilobase [kb] away from any known gene regions) were excluded. We kept only variants mapped to regulatory regions.

To preserve the familial structure, a permutation-of-residuals procedure was applied for the 1000 permutations [15,16]. First, we fitted a mixed effects linear model on the phenotypic data with all predictors in the model (except for genotype term) and preserve the residuals for these models. Second, we shuffled the residuals (rather than the phenotypic data used in an ordinary permutation procedure) and randomly assigned them to each subject and generated 1000 phenotypic data replicates. And third, we obtained the permuted statistics and *p *values by fitting a univariate linear model with genotype as the only predictor of the residuals. This method may introduce potential bias to the permuted statistics and *p *values comparing to directly fitting the full model. To quantify this potential bias, we randomly chose 1429 variants and calculated the percentage difference of the −log10 scaled *p *values obtained from directly fitting a full model and from the 2-step permutation procedure proposed in this paper.

Genotypes were coded as dominant, that is, the genotypes with 1 or 2 minor alleles were coded as 1, while genotypes with 2 major alleles were assigned 0. Variants with minor allele frequency >0.3 in genome-wide association data set were selected for PCA. We used Eigenstrat 3.0 for this analysis [17]. The R package kinship2 (http://cran.r-project.org/web/packages/kinship2/index.html) was used to calculate the kinship coefficient matrix for our data set. The R package coxme (http://cran.r-project.org/web/packages/coxme/index.html) was implemented for fitting the mixed linear model. The R package SKAT (http://cran.r-project.org/web/packages/SKAT/index.html) was implemented for rare variant analysis with SKAT. The R source code for famSKAT was downloaded (http://www.bumc.bu.edu/linga/research/publications/famskat/) and implemented for rare variant analysis. Receiver operating characteristic (ROC) curves were made and compared among the 4 algorithms and two standard methods.

## Results

**Figure 1:**
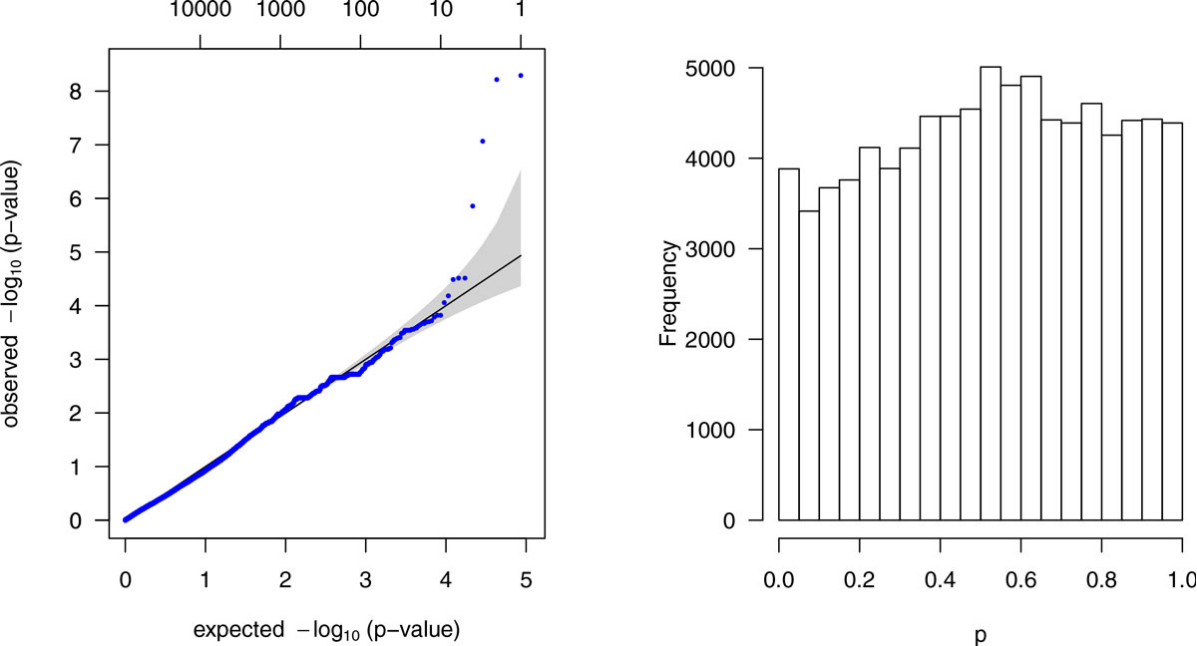
**Q-Q plot and histogram for the mixed effects model**. Q-Q plot (*left*) of −log10 scaled *p *values and histogram (*right*) for the mixed effects model based on 1237 genes (87,190 variants) from 849 subjects. In Q-Q plot, black line, expected; blue dots, observed.

**Figure 2:**
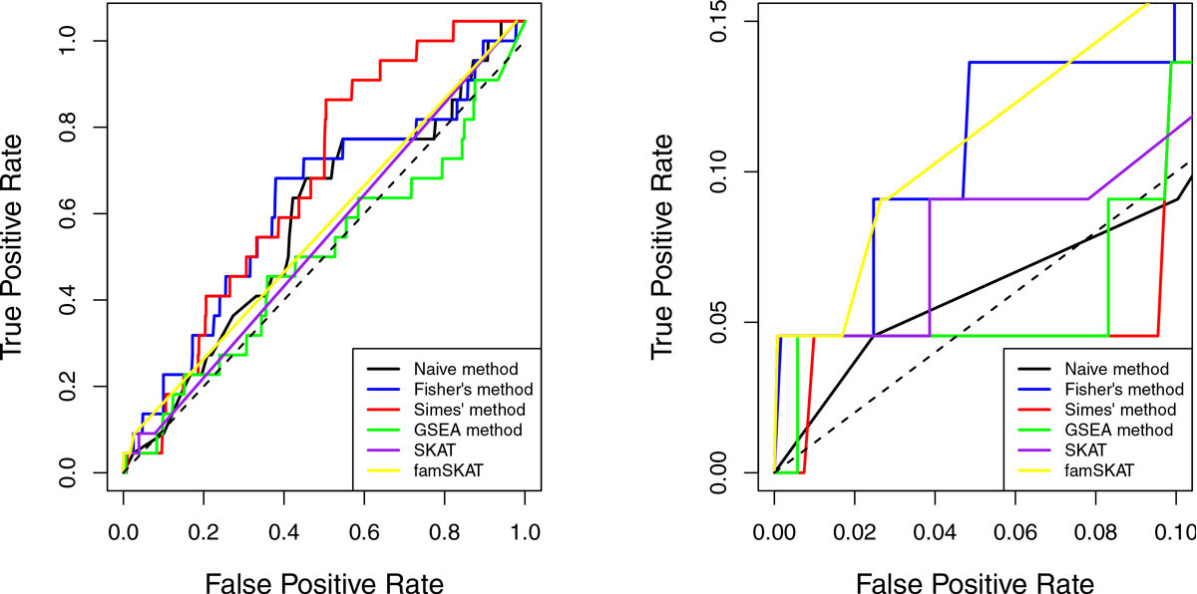
**ROC curves for 4 noncollapsing algorithms and 2 standard methods**. ROC curves for 4 different pathway algorithms based on 1237 genes from 849 subjects on trait SBP (first visit). In the left plot, false-positive rate (FPR) ranges from 0 to 1. In the right plot, FPR is scaled to be less than 0.1 as only the true-positive rate (TPR) with a low FPR is of interest. Black curve, naïve method; blue curve, Fisher's method; red curve, Simes' method; green curve, GSEA method; purple curve, SKAT; yellow curve, famSKAT.

The data consisted of 1237 genes with 87,190 variants that passed the annotation criteria were extracted from the sequencing data set of chromosome 3 for 849 subjects. After fitting the mixed linear model, the Q-Q plot and histogram of *p *values of these 87,190 variants is shown in Figure 1. To compare the 4 noncollapsing methods and the 2 standard methods, ROC curves based on these 6 methods were calculated and are shown in Figure 2.

Data for the 22 true-positive genes with 1098 variants were extracted and used for analysis with 200 simulated phenotype replicates. The statistical power information for the 6 methods was summarized and is presented in Table 1. The results of the permutation bias analysis showed that the percentage difference was only approximately 10%, and the correlation coefficient of variant-wise statistics was 0.9959. These results indicate that the effects of this bias will be limited.

**Table 1:** Comparison of the power of the 4 noncollapsing and 2 standard methods


**Chromosome**	**Gene**	**Power of methods**					
		
		**Naïve method**	**Fisher's method**	**Simes' method**	**GSEA method**	**SKAT**	**FamSKAT**

3	*ABTB1*	0.015	**0.18**	0.025	0	0.075	0.01
3	*ARHGEF3*	0	0	0	**0.035**	0.005	0.005
3	*B4GALT4*	0.015	0	0.015	**0.035**	0.01	0.015
3	*BTD*	0	0	0	**0.015**	0	0
3	*CXCR6*	0	0	0	**0.085**	0	0
3	*DNASE1L3*	0.005	0.005	0.005	0.005	0.04	**0.01**
3	*FBLN2*	0.005	0	0	**0.035**	0	0
3	*FLNB*	0.01	0.015	0	**0.03**	0	0
3	*LOC152217*	0.09	0.145	0.135	0	**0.275**	0.04
3	*MAP4*	**1**	**1**	**1**	**1**	**1**	**1**
3	*NMNAT3*	0.005	**0.04**	0.005	0	0	0
3	*PAK2*	**0.07**	0	0.05	0	0	0
3	*PDCD6IP*	0.005	0	0.005	0.005	0.04	**0.03**
3	*PPP2R3A*	**0.045**	0.01	0.02	0	0.005	0.005
3	*PTPLB*	0	0	0	**0.02**	0.005	0
3	*SCAP*	0.025	0.005	0.04	0	0.045	**0.065**
3	*SEMA3F*	0	0	0	0	0	0
3	*SENP5*	0	0.02	0.01	**0.045**	0.01	0.005
3	*SUMF1*	**0.085**	0.005	0.06	0.01	0.015	0.005
3	*TFDP2*	0	0	0	**0.035**	0	0
3	*TUSC2*	0.005	0	**0.055**	0	0.02	0
3	*ZBTB38*	0.01	0.005	0.01	0.02	**0.04**	0

Power is calculated based on the analysis of the 200 simulated phenotypic replicates. The largest power for each gene is highlighted in bold.

## Discussion

The noncollapsing methods introduced in this paper have been broadly used in testing the significance of biological pathways in GWAS data sets [11]. When we substitute the term"pathway" in these noncollapsing algorithms for the term "gene" in sequencing analysis and "gene" for "variants," we can apply these noncollapsing algorithms to gene-based association detection through modifications. An obvious advantage of aggregating *p *values (or statistics) by applying noncollapsing algorithms, compared to ordinary variants collapsing methods, is that it is a method free of the assumption that all the causal variants from a gene have effects in the same direction. This assumption may not be held in many scenarios even though it is the assumed in many existing rare variants association mapping procedures.

Another advantage of this research is the utilization of residuals-of-permutation procedure [15,16]. Conducting a permutation on family data has been a challenge in statistical genetics research. Ordinary permutation procedures have been mostly utilized in case-control data, which simply shuffle the phenotypic data and randomly assigns them to each subject, thus cannot be directly applied to family data because it destroys the family structure. In this paper, instead of shuffling the phenotypic data, we shuffled the residuals obtained from fitting a linear mixed effects model without genotype. These residuals have already accounted familial relatedness in the model fitting step and therefore our permutation procedure preserves the familial structure.

From the ROC curves in Figure 2 we note that, overall, the Simes' method performed a little better than the other 5 methods, and that GSEA, SKAT, and famSKAT did not perform as well. The other 2 methods were slightly better than the GSEA, SKAT, and famSKAT methods. However, when we limit the false-positive rate to be smaller than 0.1, as shown in the right hand plot of Figure 2 (in practice, only a high true-positive rate with a low false-positive rate is of interest), we see that Fisher's method and famSKAT performed better than the other methods at the low false-positive rate range. They both capture approximately 15% of the causal genes (true positives) at a cost of only 5% false-positive signals. However, we did not test the significance of the ROC curves, so that all these observed differences could just be noise.

From the power analysis results in Table 1, we see that the gene *MAP4 *was successfully identified to be significant for all simulated 200 replicates. All six methods achieved 100% power for this gene. This result is reasonable because, according to the "answer sheet", *MAP4 *has the most "causal variants" and these variants have a relatively larger effect size comparing to the variants within other genes. However, this result was obtained when we only analyzed 22 genes. For a genome-scale analysis, the significant signals may be missed as a consequence of correction for multiple comparisons. We have analyzed the whole genotypic data set of chromosome 3 with simulated phenotypic replicate number 1 (1237 genes and 87,190 variants). The result indicated that only naïve method and the 2 standard methods identified gene *MAP4 *to be significant. For the other 21 genes, the largest power was 0.275, which was achieved by SKAT for *LOC152217*.

Several previous researchers have already applied the noncollapsing methods proposed in this paper to conduct gene-based analysis [18,19]. However, this previous work has mainly focused on common variants in GWAS data set. As an attempt to apply these noncollapsing algorithms to gene-based association tests using sequencing data, we have demonstrated some potentially promising aspects of this approach. However, several problems remain unaddressed. One important issue is the computational intensity. In this study, we have utilized a multiprocessor computing server with a 23 × 2.8 GHz CPU and 64GB of memory. The most time-consuming part of our analysis is the permutation-of-residuals process and linear model fitting of the permuted data sets. We have paralleled this process into 20 jobs, but it still takes around 30 hours to complete (this is only the work done for 1 chromosome).

Compared to the permutation process, the *p *value combination step can be completed much faster (~30 minutes). Because a lot of the non collapsing algorithms require permutation procedures to create null distribution of the statistics, it is somewhat difficult to implement them on a genome-wide-scale data set. In addition, many noncollapsing algorithms cannot be utilized for a gene-based association test directly without proper modifications. The choice of parameters in noncollapsing algorithm for rare variant association detection is more an art than a science. Finally, adjustment for multiple hypothesis testing is another important issue that needs to be addressed.

Our results indicate that the FWER method is too conservative. For the future work, hierarchical modeling combined with the Markov chain Monte Carlo method may provide better solution to the multiple hypothesis testing problems [20].

## Conclusions

Our findings suggest that all the four new methods we proposed along with the standard method implemented with famSKAT were poor in statistical power. In sum, more research is still needed in the statistical method of association mapping for rare variants in the future.

## Competing interests

The authors declare that they have no competing interests.

## Authors' contributions

TXZ and JPR designed the overall study. TXZ and YRX conducted statistical analyses. TXZ and YRX drafted the manuscript. All authors read and approved the final manuscript. TXZ and JPR revised the manuscript critically.
